# Persona pedagogica in crisis: are educators becoming data custodians in the age of AI?

**DOI:** 10.3389/frai.2025.1743016

**Published:** 2026-01-09

**Authors:** Anusree Ambady, Thomas K. V

**Affiliations:** 1 Department of Commerce, Marian College Kuttikkanam Autonomous, Kuttikkanam, India; 2 BCM College, Kottayam, Kerala, India

**Keywords:** algorithm, artificial intelligence, pedagogical authorship, self-determination theory, teacher identity

## 1 Introduction

The introduction of AI-integrated technologies in educational systems have transformed traditional whiteboard and LMS based teaching to generative AI-based learning platforms. Contemporary educational institutions offer course planning software, automatic grading systems and dashboards to oversee learner performance. While most of the recent published studies focus on the promising side of AI integrated education, there is a hidden and significant transformation happening behind the surface. The educator's pedagogical authenticity and personal brand gets questioned as AI replaces majority of instructional and assessment functions. The present opinion piece intends to highlight the anonymous risk of erosion of teacher identity which happens due to the invisible creeping of AI in classrooms: the paradigm shift to a data custodian from a pedagogical leader. Data custodian is used to describe an educator whose main job role is slightly altered so that instead of creating and analyzing pedagogy, he is in charge of controlling, coordinating and adhering to AI-generated data streams. Such teachers do not actually serve the role of instructional author, but more and more become a mediator between algorithmic systems and learners: checking dashboards, certifying automated output, and ensuring that the intervention is consistent with system-based metrics. It is not the administrative position but rather a redesigning of pedagogical agency in which the decisions are limited or guided by algorithmic logic. This definition offers the conceptual point of reference to the arguments constructed in the paper. AI is a boon, but we should not forget to interrogate the psychological, relational and identity-based challenges that educators face in this transformation. It becomes essential to address these exposures to protect teaching as a human-centric profession from the algorithmic surroundings. This paper argues that the psychological and professional core of teaching, which is teacher identity, is at risk of erosion. Rooted in Self-Determination Theory, this article explores how AI challenges teachers' autonomy, competence and relatedness.

## 2 Manuscript formatting

### 2.1 Conceptual and methodological orientation

Grounded in a qualitative review of existing literature, this paper employs a conceptual and interpretive approach to examine how the integration of AI in education settings influences teachers' pedagogical identity. The interpretations and insights of this paper are synthesized from peer-reviewed Scopus-indexed studies, rather than reporting primary empirical data. The works considered eligible for this study are papers focusing on teacher autonomy, professional identity and the psychological implications of automation in an educational perspective. Significant articles were identified using databases like Scopus and Web of Science, using keywords including “teacher identity,” “AI in education,” “algorithmic decision making,” and “data-driven pedagogy.” The studies that expressly discussed the AI oriented teaching practices and teachers' emotional responses to technological adoption were considered for this opinion article. The study followed a thematic approach in identifying ongoing patterns in the literature based on connections between SDT, algorithmic mediation and pedagogical identity erosion. The study also examines how AI affects educators' autonomy, competence and relatedness, which are key dimensions that shape professional identity. Considering the nature of the topic, this paper magnifies critical reflection on statistical generalization. It also paves the way for future empirical studies uncovering teacher identity and agency in AI-driven educational environments.

### 2.2 Theoretical framework and background

The concept of teacher identity relies on the sense of self by combining personal emotions, values and beliefs with professional knowledge, institutional and pedagogical practices. This construct is shaped through reflection, interaction and adaptation to changing educational contexts (Beauchamp and Thomas, 2009; Pennington and Richards, 2016). Self-Determination Theory (SDT) (Deci and Ryan, 2000) gives a solid psychological framework for explaining how teacher identity is formed, sustained or disrupted in such contexts. As per the theory, human motivation and wellbeing depend on the fulfillment of three basic psychological needs: autonomy, competence and relatedness (Brenner, 2022; Deci et al., 2017; Gagné and Deci, 2005). Satisfaction of these needs makes teachers experience self-directed motivation, professional agency and a stable sense of identity; hindrance of which leads to decline in motivation and authenticity (Deci et al., 2017; Gagné and Deci, 2005). When AI mediates educational environments, teachers' autonomy may be blocked by algorithmic systems, competence challenged by technological dependence and relatedness is weakened by reduced human interaction. This frustration of psychological needs can disrupt professional identity and diminish pedagogical commitment. Thus, SDT offers a valuable lens for interpreting how the poor coordination of AI in automation of teaching tasks may erode authenticity, motivation and resilience at the core of teacher identity (Lan, 2024). Hence, Self-Determination Theory provides a psychological explanation within AI-mediated educational environments on how automation may impair the intrinsic needs sustaining teachers' professional identity.

### 2.3 From pedagogical authority to algorithmic mediation

Conventionally, teaching is comprised of designing the learning experiences, suiting the student needs and ethical analysis of classroom interactions. With the integration of AI, algorithmic logic dominates many of these decisions—what content to deliver, who needs feedback, and what the next exercise should be. Studies prove that educators do use AI tools for content adaptation, assessment support and feedback loops at high rates (Lan, 2024). A review study shows that many studies focus on teachers' behaviors, perceptions, and digital competence regarding data-driven tools (Salas-Pilco et al., 2022). The data sources are often behavioral, discourse, or statistical data, analyzed through algorithms. These expectations place teachers in roles of collecting, interpreting and acting on data. AI tools help teachers in relieving repetitive tasks, but they also constrain teacher autonomy. For example, in a professional development program in Turkey, which specifies that while teachers appreciate tools that reduce workload, they also feel that AI-supported systems prescribe certain content delivery pace or standardized workflows (Filiz et al., 2025). Similarly, algorithmic recommendations and metrics-based protocols can limit teachers' decisions, potentially conflicting with their pedagogical values (Küçükuncular and Ertugan, 2025). Students notice the changes in teacher authority, care and expertise when AI is integrated in the classroom. Some students want their teachers to be more of a mentor rather than a content provider (Almashour et al., 2025). The evolving expectations of learners push teachers to adjust their teaching methods, especially in AI and data driven educational environment. This subtle but significant shift in the teacher's role from originator to implementor of algorithmic routes, it alters the locus of pedagogical authority. Teachers might focus on monitoring the data outputs rather than initiating them. Gradually, the teaching profession may be aligned with more of data governance rather than teaching leadership.

Although these studies map out the adoptions of AI, the issue is more than technical adoption to subtle restructuring of power in the classroom. The increasing reliance on algorithmic signals reinstatement of pedagogical authority as a system output, as opposed to professional agency. According to this opinion, this reorganization is not a neutral rearrangement but a radical repositioning of teachers as actors in the data ecosystems, reducing the purposive, human-focused quality of pedagogy.

### 2.4 The psychological cost of automation

Beyond a functional role, professional identity is a source of purpose, belonging and self-efficacy for teachers. When educators lose a sense of authorship over content, feedback, and instructional design, they may experience instructional detachment: teaching becomes monitoring rather than meaningful facilitation. The transition to data custodianship is aligned with the psychological needs that are at the heart of the Self-Determination Theory. Under the condition teachers are mostly supervisors of algorithmic recommendations, their agency is limited since the choice of instruction is limited to a set system trajectory. The issue of competence is influenced to the extent that their professional judgment is dwarfed by the automated decision-making, which brings about the reliance to the technological products instead of professional judgment. The relational feedback is obstructed by the use of data-driven tools, which substitute relational with system generated cues. This three-part interference offers a psychological rationale as to why the data custodian position puts the teacher identity at risk. The report of a qualitative investigation conducted in AI-rich classroom environments, shows that students noted that teachers' authority was fading even though human relational presence was still valued (Almashour et al., 2025). These findings illustrate that efficiency comes at the cost of diminished pedagogical agency, a theme that recurs across AI-mediated educational research.

Studies do support this concern (Filiz et al., 2025). Found that beyond the deployment of new technologies in the classrooms, educators readjust their professional values and sense of agency in the classroom. In research on teacher leadership roles, authors stated that teachers' autonomy is weakened by AI algorithms, thus turning them into implementers rather than innovators (Ghamrawi et al., 2024). This reduction in agency and leadership opportunity can lead to diminished professional pride and identity.

### 2.5 Drivers of the shift to data custodianship and associated risks for teachers and pedagogy

Building on this conceptualization, the following section outlines the structural and technological forces that actively shape teachers into data custodians, along with the pedagogical risks such a shift entails (Table 1).

**Table 1:** Drivers and risks associated with data custodianship.

**Drivers of the shift to data custodianship**	**Risks/Consequences for teachers and pedagogy**
Algorithm-based lesson design	Teachers become implementers rather than designers as they lose pedagogical creativity and ownership over the instructional framework
Automated assessment and feedback tools	Weaken teacher-student connection as it diminish relational feedback
Student performance dashboards	Prioritizing metrics over meaning; teachers feel like data monitors
Administrative automation	Role ambiguity and professional deskilling
Institutional accountability systems prioritize measurable outputs	Techno pressure leads to burnout, reduced autonomy and loss of intrinsic motivation
AI mediated student interactions	Students rely more on AI feedback than teacher guidance

Source: Author's compilation based on reviewed literature.

### 2.6 Discussion

Teachers are increasingly being asked to serve as data custodians. But the erosion of pedagogical identity or transformation heavily depends on how the change is managed. There are several ways to prevent erosion. Ensure participation of educators in designing, selecting and deploying AI and analytical tools in classrooms. Teachers can be trained on how to integrate AI in data literacy, interpretative judgement and critical engagement by preserving their spaces to reflect on how data tools align or conflict with their values. Accountability should not be wholly relied on measurable outputs. Rather, qualitative, relational, process-oriented and ethical aspects of teaching should be considered in evaluation. Proper understanding should be ensured on the working of AI algorithms, the nature of data, potential biases and the implications for students and teachers. Systems must safeguard privacy, spotlight uncertainty, allow teacher intervention in decision making and avoid incites. The relational, empathetic and moral side of teaching-learning must be guarded amidst AI data tools. This helps in protecting teacher identity as well as students' path of learning that metrics can't fully capture. Pragmatically, by constructing clear mechanisms of identity erosion, institutions may reduce identity erosion by creating explicit points of human decision-making in AI-aided processes, so that the eventual instructional judgement does not rest with the artificial intelligence. Schools can use co-design procedures whereby teachers consider the pedagogical suitability of algorithmic suggestions prior to their adoption. Also, there are more formal forums of reflective dialogue, such as AI ethics circles or professional learning communities, which might assist teachers in formulating conflicts between data-guided and value-guided practice. This kind of measures shifts the discussion on hypothetical risks to strategies that can be put in place.

### 2.7 Scope for further research

This article offered a conceptual perspective on the erosion of pedagogical identity in AI-surrounded contexts. Several opportunities remain open for empirical and theoretical exploration. Further studies could empirically validate the concept of instructional detachment across school and higher education settings. Cross-cultural comparisons of teacher identity in AI-integrated systems would reveal how identity is shaped by sociocultural values, accountability pressures and AI adoption maturity. Future studies may research how automation affects emotional labor, job satisfaction and burnout, especially in an occupational psychology background. Design-based research focusing on AI integration that explicitly aids teacher creativity and pedagogical authorship is still untouched. Exploration of these domains through interdisciplinary research can help in creating an educational future where AI can strengthen teachers' agency and professional identity.
